# Lead Tolerance and Accumulation in *Hirschfeldia incana,* a Mediterranean *Brassicaceae* from Metalliferous Mine Spoils

**DOI:** 10.1371/journal.pone.0061932

**Published:** 2013-05-07

**Authors:** Florence Auguy, Mouna Fahr, Patricia Moulin, Anaïs Brugel, Laurent Laplaze, Mohamed El Mzibri, Abdelkarim Filali-Maltouf, Patrick Doumas, Abdelaziz Smouni

**Affiliations:** 1 Institut de Recherche pour le Développement, Unité Mixte de Recherche Diversité Adaptation et Développement des Plantes, Montpellier, France; 2 Centre National de l’Energie, des Sciences et des Techniques Nucléaires, Laboratoire de Biotechnologie des Plantes, Rabat, Maroc; 3 Laboratoire Mixte International, Université Mohammed V - Agdal, Rabat, Maroc; 4 Laboratoire de Physiologie et Biotechnologie Végétale, Université Mohammed V - Agdal, Rabat, Maroc; 5 Institut de Recherche pour le Développement, Unité de Service Instrumentation, Moyens Analytiques, Observatoires en Géophysique et Océanographie, Laboratoire de Microbiologie et Biologie Moléculaire, Université Mohammed V - Agdal, Rabat, Maroc; 6 Institut de Recherche pour le Développement, Laboratoire mixte international Adaptation des Plantes et microorganismes associés aux Stress Environnementaux, Laboratoire Commun de Microbiologie, Dakar, Sénégal; 7 Laboratoire de Microbiologie et Biologie Moléculaire, Université Mohammed V - Agdal, Rabat, Maroc; 8 Institut National de la Recherche Agronomique, Unité Mixte de Recherche Biochimie et Physiologie Moléculaire des Plantes, Montpellier, France; Iwate University, Japan

## Abstract

Lead is a heavy metal of particular concern with respect to environmental quality and health. The lack of plant species that accumulate and tolerate Pb is a limiting factor to understand the molecular mechanisms involved in Pb tolerance. In this study we identified *Hirschfeldia incana*, a *Brassicaceae* collected from metalliferous mine spoils in Morocco, as a Pb accumulator plant. *H. incana* exhibited high Pb accumulation in mine soils and in hydroponic cultures. Major Pb accumulation occurred in the roots and a part of Pb translocated from the roots to the shoots, even to the siliques. These findings demonstrated that *H. incana* is a Pb accumulator species. The expression of several candidate genes after Pb-exposure was measured by quantitative PCR and two of them, *HiHMA4* and *HiMT2a*, coding respectively for a P1B-type ATPase and a metallothionein, were particularly induced by Pb-exposure in both roots and leaves. The functional characterization of *HiHMA4* and *HiMT2a* was achieved using Arabidopsis T-DNA insertional mutants. Pb content and primary root growth analysis confirmed the role of these two genes in Pb tolerance and accumulation. *H. incana* could be considered as a good experimental model to identify genes involved in lead tolerance and accumulation in plants.

## Introduction

Lead (Pb) is a heavy metal of particular concern with respect to environmental quality and health [1]. As a non-essential trace metal for biological functions, it is highly toxic to plants and animals. Sources of anthropogenic soil contamination by Pb include industrial and agricultural activities such as mining and smelting of metalliferous ores, battery-engine waste, wastewater irrigation, and overuse of chemical fertilizers and pesticides [2]. Therefore, the cleanup of Pb-contaminated soils is imperative. In this context, phytoremediation can be considered as a potent tool in the near future. Phytoextraction is based on the genetic and physiological capacity of specialized plants to tolerate high amounts of metal, to translocate from roots to shoots, and to accumulate in shoots [3]. The idea of using plants to remove metals from soils came from the discovery of different wild plants that accumulate high concentrations of metals in their foliage [4]. Currently, phytoremediation of Pb-polluted soils presents two major drawbacks namely, on the one hand, the limited number of species which hyperaccumulate Pb and, on the other hand, the insufficient knowledge of the molecular mechanisms implicated in Pb tolerance in plants.

Over 450 species of metal hyperaccumulator and tolerant plants are known, in both tropical and temperate zones, and most of them are nickel hyperaccumulators [5], [6]. Plant species which hyperaccumulate cobalt, copper or zinc are in second rank, although in much smaller numbers. Hyperaccumulation of arsenic, cadmium, gold, lead, manganese and thallium occur in a limited number of species [7], [8]. Lead accumulating species are rather exceptional. It was well established by Baker *et al.* [4] that *Noccaea caerulescens* can accumulate Pb to high concentrations from nutrient solutions with low concentration of added Pb, mostly fixed in the root with limited translocation to the leaves. Few other species have been described as Pb hyperaccumulators such as *Agrostis tenuis*, *Festuca ovina*, *Rumex acetosa* or *Thlaspi cepaeifolium* [9].

Concerning the molecular elements implicated in lead-tolerance mechanisms, the number of identified genes is quite small. The tobacco plasma membrane protein NtCBP4 and the Arabidopsis gene *CNGC1* were reported to be components of a transport pathway responsible for Pb entry into plant cells [10]. An Arabidopsis P-type ATPase, HMA3, was described to improve tolerance by sequestrating Pb in the vacuole [11], [12]. HMA4, another P-type ATPase, from *Noccaea caerulescens* has also been suggested as having a potential role in Pb efflux transport in yeast [13]. In Arabidopsis, three members of ABC (ATPase-binding cassette) transporters family AtATM3, AtPDR12 and AtPDR8 contribute to Pb resistance [1], [14], [15]. Recently, ACBP1, an acyl-CoA-binding protein, was found to be involved in mediating Pb tolerance through accumulation of Pb in shoots [16] and *AtMRP3* transcription was also shown to be strongly induced by Pb treatment [17] in *A. thaliana*. An important mechanism controlling heavy metal tolerance is chelation that involves small molecules such as metallothioneins, phytochelatins and glutathione [18], [19].

Here, we identified *Hirschfeldia incana*, a member of the *Brassicaceae* family, collected in abandoned lead mining sites in the east of Morocco, as a Pb accumulator plant and we demonstrated that this species, with a close genetic proximity to Arabidopsis, is a good experimental model to identify genes involved in lead tolerance and accumulation in higher plants.

## Material and Methods

### Ethics Statement

No specific permits were required for the described field studies because sample collection does not involve any endangered or protected plant species or privately-owned locations.

### Plant Material and Growth Conditions

Seeds of *H. incana* were harvested from plants growing in abandoned metalliferous mine spoils in the mine district of Oued El Himer, south of Oujda city in eastern Morocco (34°26′88′′N, 1°54′03′′W; Smouni *et al.*, 2010). Seeds were surface sterilized, sown on one half MS medium [20] with 1.2% agar and grown vertically in a growth chamber at 22°C with a photoperiod of 16 h of light (0.1 mMol.m^−2^.s^−1^) from fluorescent lamps. After 2 weeks, seedlings were transferred to hydroponic culture on BD medium [21] containing 5 mM KNO_3_, 1 mM CaCl_2_, 0.5 mM KH_2_PO_4_, 0.25 mM MgSO4, 0.25 mM K2SO4, 1 µM MnSO4, 50 µM FeEDTA, 2 µM H_3_BO_3_, 0.5 µM ZNSO_4_, 0.2 µM CuSO_4_, 0.1 µM CoSO_4_ and 0.1 µM Na_2_MoO_4_. Lead treatment was done by adding 50, 100 or 300 µM Pb(NO_3_)_2_ to a fresh BD medium without phosphate to avoid Pb precipitation. For the cultivation of *H. incana* on different soils with various lead concentrations, seedlings were grown on sterile compost for 20 days and transferred to four different soils from metalliferous mine spoils containing various amounts of lead (soil 1∶6972 mg.kg^−1^ DW, soil 2∶18626 mg.kg^−1^ DW, soil 3∶7531 mg.kg^−1^ DW and soil 4∶1577 mg.kg^−1^ DW; table 1).

**Table 1:** Pb levels of the metalliferous mine soils in the plant cultivation.

Fractions	Soil 1	Soil 2	Soil 3	Soil 4
Total Pb (mg/Kg)	6972.8±59.4	18626.2±125.1	7531.1±86.2	1577.2±36.7
10 mM CaCl2 extractable Pb (mg/Kg)	5.8±0.1	2.8±0.2	1.2±0.1	1.5±0.3
50 mM EDTA extractable Pb (mg/Kg)	6665.9±195.4	18315.8±268.1	7166.8±822.3	1445.6±72.2

Pb-polluted soils were collected in different zones from abandoned metalliferous mines of Oued El Himer, south of Oujda city in eastern Morocco. Data are the average (± SE) of three independent measurements.

*Arabidopsis thaliana* ecotype Columbia (Col-0) and T-DNA mutant seeds were obtained from the Nottingham Arabidopsis Stock Centre [22]. Seeds were surface-sterilized and sown on square Petri dishes containing BD medium without phosphate, with 1.2% agar, with or without 40 µM Pb(NO_3_)_2_. After sowing on either standard or Pb-treated medium, seeds were cold-treated at 4°C for 48 h in darkness to promote and synchronize germination, subsequently transferred in a vertical position to a growth chamber at 22°C with a photoperiod of 16 h of light (0.1 mMol.m^−2^.s^−1^). In hydroponic cultures, *A. thaliana* Col-0 seeds were prepared as described previously for *H. incana* seeds and seedlings were transferred after two weeks on liquid BD medium. The Pb-treatment was done by adding 40 µM Pb(NO_3_)_2_ to a fresh BD medium without phosphate.

### Lead Quantification

Shoots or roots (three independent replicates per sample) were washed twice in cold 0.2 mM CaSO_4_ and rinsed with cold distilled water. For the roots, rinsing with water and CaSO_4_ does not remove adsorbed Pb, and hence all root tissue Pb measurements reported in this study include both the Pb taken up by the plant and the Pb adsorbed at the root surface. Samples were dried at 72°C for 48 h and a maximum of 200 mg of dried tissues were treated according to the acid hydrolysis protocol described by Temminghoff and Houba [23]. Soil samples were air-dried at 70°C to constant weight. Dried soils were sieved through a 2 mm mesh and ground in a porcelain pestle and mortar. To estimate total lead amount from each prepared soil, 0.5 g (three replicates per sample) was treated as described by Smouni *et al.* [24]. The mobile and mobilisable fractions in the soils were estimated by using 10 mM CaCl_2_ and 50 mM EDTA at pH 7, respectively. Two grams of soil samples (three replicates per sample) were suspending in 20 mL of 10 mM CaCl_2_ or 50 mM EDTA and shacked for two hours. The suspensions were centrifuged at 8000 rpm for 12 min and lead concentration was analyzed in the supernatants. Lead concentration was determined by ICP-AES (Inductively Coupled Plasma-Atomic Emission Spectrometry; Ultima2 JY) at a wavelength of 220.353 nm in accordance with the method devised by Margui *et al.* [25].

### DNA Extraction

Hundred mg of fresh leaf tissue of *H. incana* were harvested directly in 500 µL of extraction buffer (100 mM TrisHCl, 500 mM NaCl, 50 mM EDTA, pH 8.0) and 70 µL of SDS 10% (w/v) were added. Samples were homogenised, incubated 10 min at 65°C and 130 µl of 5M potassium acetate were added. After homogenisation, samples were centrifuged 15 min at 13 000 rpm and 500 µL of the supernatant were added to 500 µL of propanol 2. The precipated DNA was recovered by centrifugation 5 min at 13 000 rpm. The pellet was dried and resuspended in 30 µL of ultra pure water. DNA extraction was done on three independent replicate experiments.

### RNA Extraction

Total RNA was extracted from 100 mg of frozen samples, using the RNeasy Plant mini kit (Qiagen, USA) for roots and the SV Total RNA isolation system (Promega, USA) for shoots. All samples were DNase treated using the Turbo DNA-free (Ambion, USA) in accordance with the manufacturer’s protocol. The RNA quality was confirmed by non-denaturating electrophoresis. RNA extraction was done on three independent replicate experiments.

### Quantitative Real-time Reverse-transcription PCR

First-strand synthesis was carried out using 1 µg of total RNA with the reverse transcription system (Promega, USA) and oligo(dT)_20_ primers. For the three biological replicates, two independent reverse transcriptions were done and pooled to minimize variation in reverse transcription yield. An equal amount of cDNA was used for each reaction, corresponding to 1.5 ng of total RNA. A reaction contained 7.5 µL of Brillant II Sybr Green QPCR master mix (Agilent, USA), 2.25 pmol of each primer in a total volume of 15 µL. The reactions were done on the MX3005P apparatus (Agilent, USA) under the following thermal profile: 5 min at 95°C, 40 repeats of 10 s at 95°C, and 30 s at 60°C and a final stage to determine dissociation curves of 1 min at 95°C, 30 s at 55°C and after, constant temperature increasing to 95°C with the fluorescence reading in continuous. The primer set was designed with the Primer 3 software and primers are listed in table S1. The efficiency of each primer pair was determined by amplifying serial dilutions of cDNA. Tubulin gene was chosen as reference gene because there was less than 1 threshold cycle (Ct) difference among the different samples and conditions. Relative expression ratios were calculated using the comparative ΔCt method and efficiencies of each gene were taken into account. The calibration was done against roots or leaves from plants growing in standard conditions (without lead). The RNA level from the three biological samples was measured in triplicate.

### Internal Transcribed Spacer (ITS) Sequence Isolation

The complete ITS region (including ITS 1 and 2 and the 5.8 S r DNA gene) was amplified with the primers ITS4 and ITS5 [26]. PCR products of 722 bp were cloned into pGEM-T easy vector (Promega) and sequenced on both sides with the universal M13 primers. Three ITS regions were sequenced from three PCR reactions done on the three independent DNA extractions. DNA sequences were aligned with the software Clustal. Percentage identity of the *H. incana* ITS consensus sequence and *A. thaliana* ITS sequence was determined with the software Blast.

### *H. incana* Gene Cloning

PCR was achieved on roots or shoots of *H. incana* cDNA treated with 100 µM Pb(NO_3_)_2_ for 3 days. Primers were designed directly from the literature or from *A. thaliana* sequences or by alignment of multiple sequences (Table S2). PCR fragments were purified with the Wizard SV PCR Clean up system (Promega) and ligated in the pGEM-T easy vector (Promega) before transforming *E. coli* competent cells. PCR products were sequenced by automated DNA sequencing (Eurofin MWG Operon) and gene identities were confirmed by comparison with the BLAST algorithm against the Genbank sequences.

### Identification of Arabidopsis T-DNA Insertion Mutants

Multiple alignments of nucleotide sequences reveal high sequence identity with respectively HiHMA4 and At HMA4, and HiMT2a and AtMT2a. In addition, phylogenic trees point to HiHMA4 and HiMT2a as putative orthologs of respectively AtHMA4 and AtMT2a (Figure S1). Consequently, homozygote plants of the T-DNA insertion lines Salk_093482 and Salk_059712 for the genes At2g19110 (*AtHMA4*) and At3g09390 (*AtMT2a*) respectively were identified by PCR using 3 different primers. In the case of Salk_093482, a T-DNA specific primer (LBb1, 5′-GCGTGGACCGCTTGCTCAACT-3′) and two AtHMA4 specific primers (AtHMA4-F 5′-CACTTGACGGCGTTAAAGAA-3′ and AtHMA4-R 5′-AACCATGACGCAAAACCACT-3′) were used. In the case of Salk_059712, the same LBb1 T-DNA specific primer and two AtMT2a specific primers (AtMt-F 5′- CCATAACACACGGAACATCG-3′ and AtMt-R 5′-AGATCCACATCCGCAGTTT-3′) were used. The genotype of the F3 individuals was checked by PCR using gene-specific primers and T-DNA primers. Individual homozygous mutants were backcrossed twice with the wild-type Col-0.

### Root Growth Analysis

Images of the root system were acquired using a desktop scanner (with a resolution of 450 dpi) directly from Arabidopsis plants growing in Petri dishes after 13 days of culture. Images were analyzed using Optimas software version 6.1 (Media Cybernetics, MD, USA). Data were exported to an Excel worksheet for final processing.

### Statistical Analysis

All data are expressed as arithmetic means +/− SD of replicate plants within an experiment. All data shown are from one experiment representative of a total of two or three independent biological experiments. All results were statistically analyzed using Statistica software version 7.1 (Statsoft, Tulsa, OK, USA). For the analysis of variance, 2-factors ANOVA with a LSD Post Hoc test were used to measure differences (*p*<0.01).

## Results

### *H. incana* Accumulates Pb in Both Leaves and Roots

**Figure 1:**
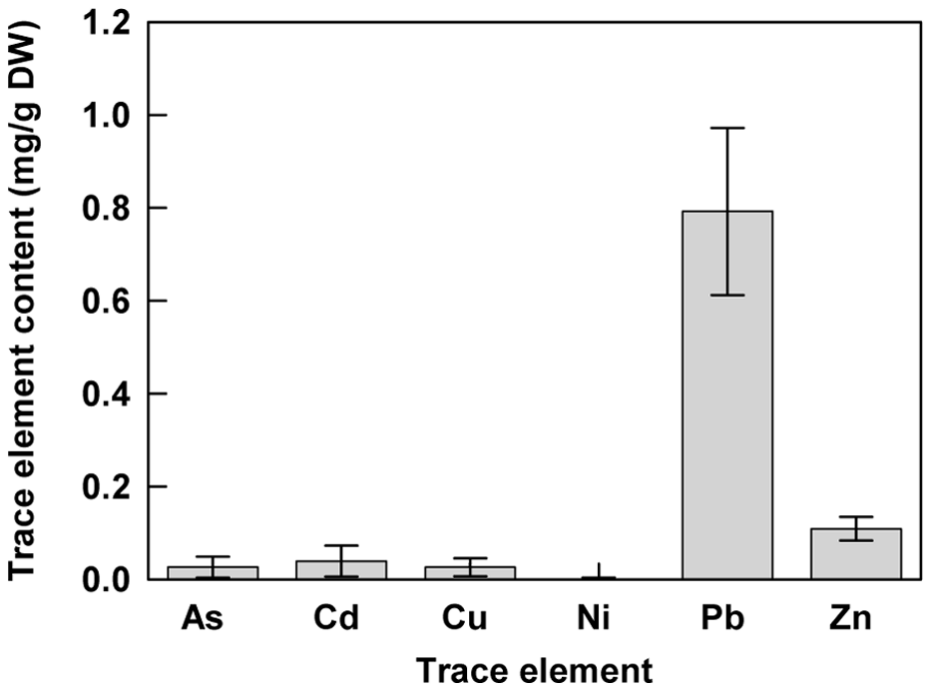
Amount of metallic trace elements (As, Cd, Cu, Ni, Pb and Zn) in shoots of *H. incana.* Plants were collected from different zones in a heavy metal-polluted area. Data are the average (± SE) of six independent measurements. Amount (average ± SE) of metallic trace elements in the soil (mg/Kg): As = 298±80; Cd = 10±4; Cu = 459±88; Ni = 13±6, Pb = 7157±746; Zn = 14164±1460.

**Figure 2:**
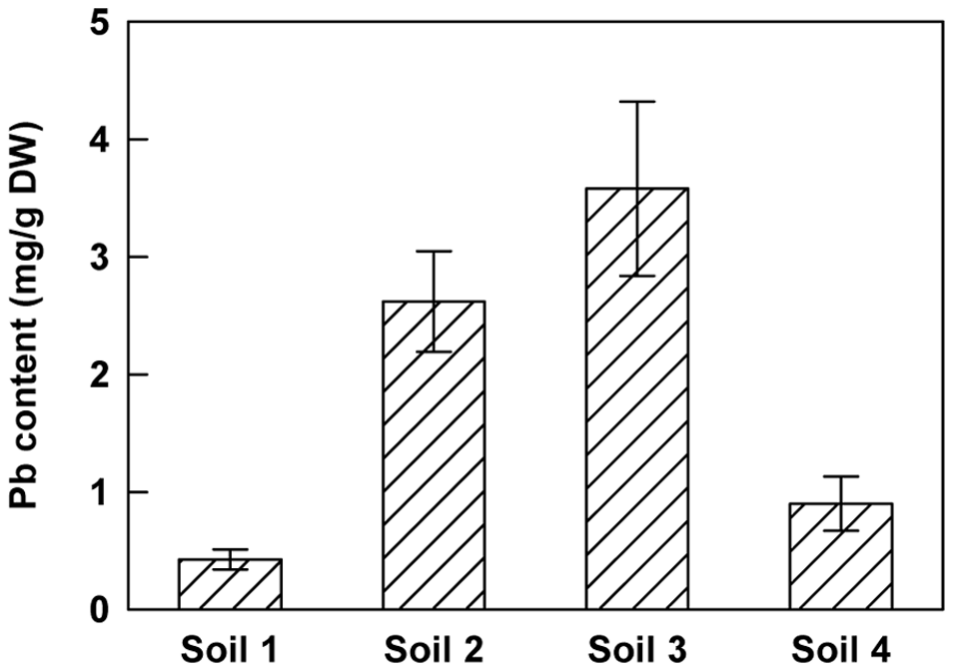
Lead concentration in shoots of *H. incana* growing on different polluted soils. Seedlings were grown on sterile compost for 20 days and transferred for 2 months on 4 different soils containing various amounts of total lead (soil 1∶6972 mg.kg^−1^ DW, soil 2∶18626 mg.kg^−1^ DW, soil 3∶7531 mg.kg^−1^ DW and soil 4∶1577 mg.kg^−1^ DW; Table 1). Data are the average (± SE) of three independent measurements.

An exploration of the flora from mining sites in Oued El Himer region, located in the south of Oujda city (Eastern Morocco), was conducted in order to identify new species able to accumulate heavy metals in their shoots for a future phytoremediation project. The sites we studied have been heavily affected by lead mining and smelting activities, and soils are subject of a polymetallic contamination [24]. In these areas, lead concentrations in soils varied from 26 to 9479 mg.kg^−1^ [24]. Plants were collected and leaf samples were analyzed by ICP-AES in order to quantify heavy metal contents. The *Brassicaceae H. incana* was particularly interesting because of its accumulation profile of toxic heavy metals in leaf tissue (Figure 1). In natural conditions, this species presented a high level of Pb in leaves ranging from 0.53 to 1.43 mg.g^−1^ DW with an average of 0.79 mg.g^−1^ DW (Figure 1). The other metals analyzed were present at lower concentration such as Cd or Zn with an average of 0.04 and 0.11 mg.g^−1^ DW respectively even if the concentrations of these metals were high in the different sampled soils [24]. These results show that *H. incana* seems to be highly specific for Pb accumulation in accordance to the contents of the different heavy metals analyzed. In order to confirm the accumulator trait observed in natural conditions and to avoid air-borne contamination such as dust deposits, *H. incana* plants were grown under controlled growth chamber conditions on four different soils collected in metalliferous mine spoils. These soils contained various amounts of total Pb (soil 1∶6973 mg.kg^−1^ DW; soil 2∶18626 mg.kg^−1^ DW; soil 3∶7531 mg.kg^−1^ DW; soil 4∶1577 mg.kg^−1^ DW, table 1). After 2 months of culture, Pb content was quantified in the shoots (Figure 2). The concentration of Pb in leaves was variable from a minimum of 0.43 mg.g^−1^ DW for plants grown on soil 1 to a maximum of 3.58 mg.g^−1^ DW for those grown on soil 3. These results confirmed the lead hyperaccumulator trait for *H. incana*. On the other hand, no correlation was observed between total Pb content in the different soils and Pb level in the plant tissues suggesting variations in the amount of bioavailable lead. CaCl2 and EDTA extractible fractions corresponding respectively to the mobile and the mobilisable fractions were quantified (Table 1). No correlation could be demonstrated between the Pb levels of the extractible fractions and those of the *H. incana* assimilated fraction.

In the light of these results and according to the standard criteria on heavy metal hyperaccumulation (i.e. >0.1% DW of Pb; [4], [8]), *H. incana* could be considered as a lead hyperaccumulator plant.

### *H. incana* Translocates Pb from Roots to Shoots

**Figure 3:**
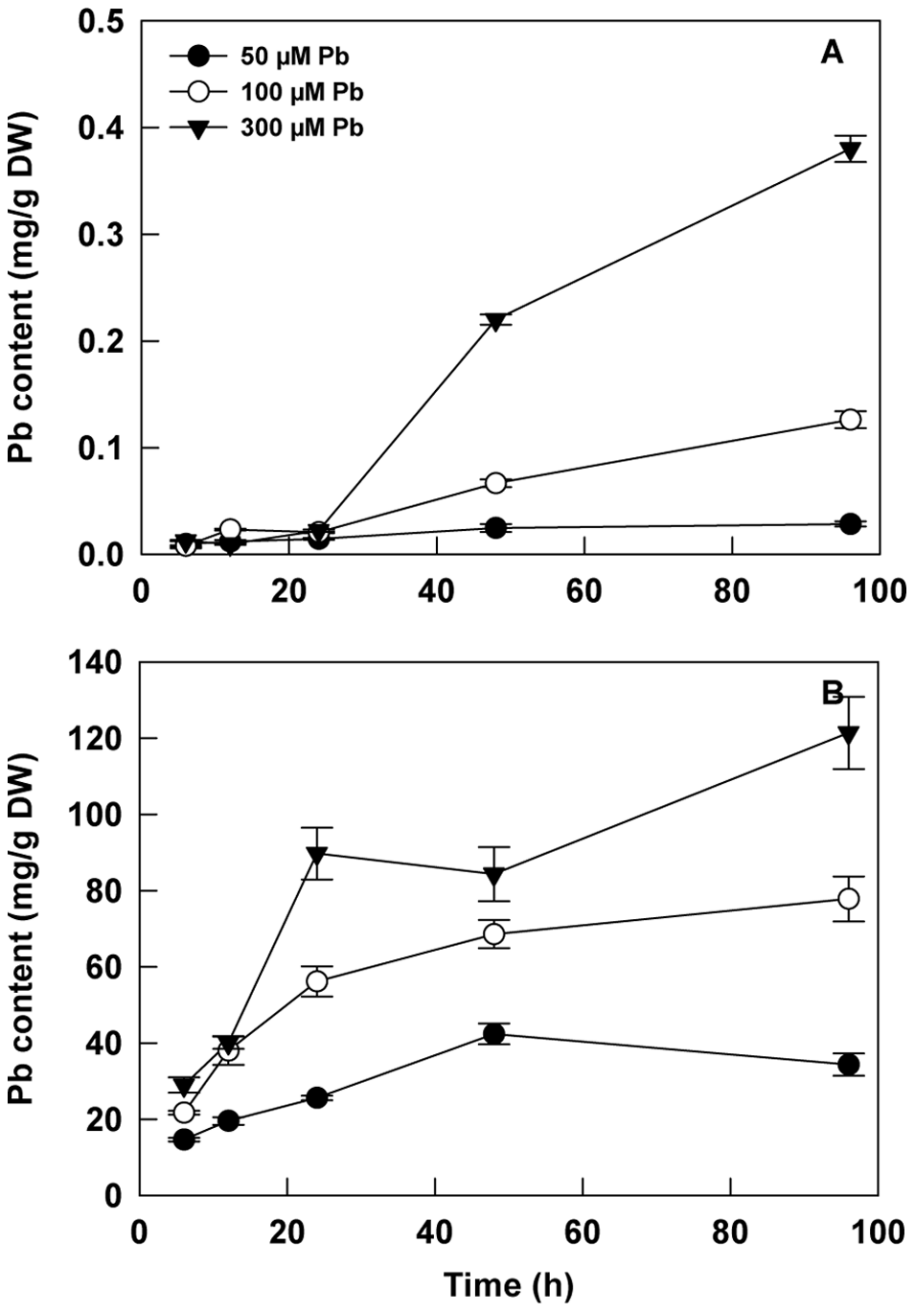
Kinetic effect of lead exposure on lead accumulation in (A) shoots and (B) roots of *H. incana*. Seedlings were grown on hydroponic system and lead treatments were done after 2 weeks by adding 50, 100 and 300 µM Pb(NO_3_)_2_ in fresh medium. Data are the average (± SE) of three independent measurements.

A four-day kinetic study, in hydroponic conditions, was achieved to confirm the transport of lead from roots to shoots in *H. incana* (Figure 3). Three Pb concentrations were tested (50, 100 and 300 µM Pb(NO_3_)_2_). In the study period and with these concentrations of Pb, no visual damage could be observed neither in leaves and roots. The profiles of lead accumulation in roots or in shoots were similar for the different concentrations with a proportional response to the Pb concentration in the medium. In hydroponic conditions, lead accumulation in roots and shoots was found to be dose and time dependent: roots showed more accumulation than shoots at the same concentration and exposure periods. In the shoots, a slow but constant increase of lead content after a latency period of 24 hours was observed for the three concentrations (Figure 3A). In the roots, the levels of Pb gradually increased with time of exposure for expected values of 42, 69 and 121 mg.g^−1^ DW for respectively 50, 100 and 300 µM of Pb in the medium. A slight decrease of the curve was observed for the last points of kinetics for the 50 µM concentration and a plateau for the 100 µM concentration (Figure 3B). Finally a maximum of translocation was observed at day-4 for 300 µM of Pb in the medium where 3% of total Pb in the plant were localized in the shoots.

**Figure 4:**
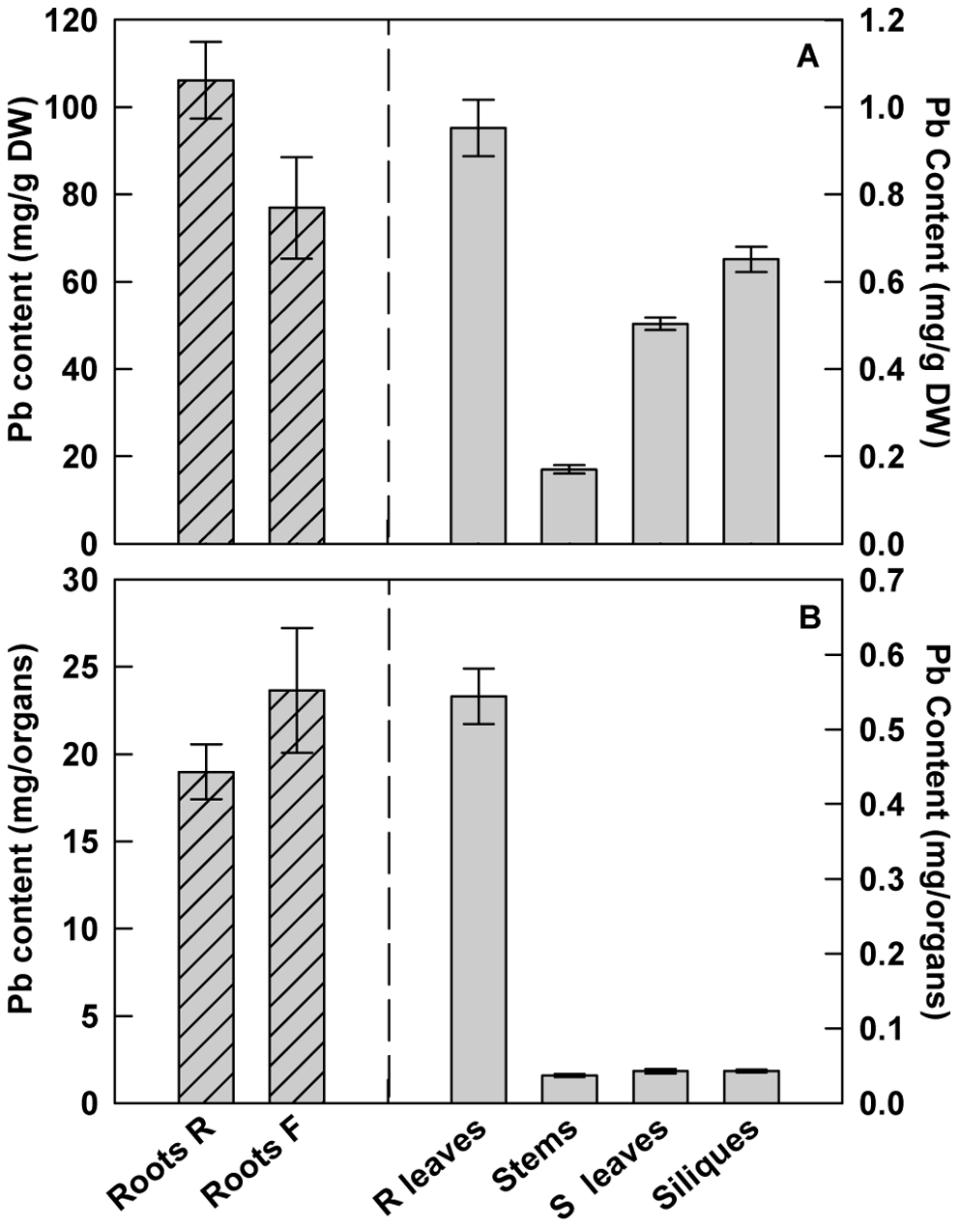
Lead distribution in different parts of *H. incana*. Lead contents are expressed as (A) mg Pb.g^−1^ DW or (B) mg Pb.organ^−1^. Seedlings were grown for 2 months in hydroponic conditions supplied weekly with fresh media containing 100 µM Pb(NO_3_)_2_. Roots R = roots at the rosette stage; Roots F = roots at the floral stage; R leaves = rosette leaves; S leaves = stem leaves. Data are the average (± SE) of three independent measurements.

In order to evaluate the distribution of Pb in different parts of the plant, *H. incana* was grown in hydroponic conditions supplied weekly with fresh medium containing 100 µM Pb(NO_3_)_2_. After 2 months of Pb exposure, *H incana* was able to accumulate up to 106 and 77 mg Pb.g^−1^ DW in roots at the vegetative stage and in roots at the floral stage respectively (Figure 4A). The aerial plant organ with the highest Pb concentration was the rosette leaves reaching up to 0.95 mg.g^−1^ DW following by the siliques where the Pb content may reach 0.65 mg.g^−1^ DW. When data were expressed per organ (Figure 4B), strong accumulation of Pb was also observed in the roots (19 and 23.7 mg Pb in roots at the vegetative stage and in roots at the floral stage respectively) but a significant presence of Pb has also been noted in the shoots (0.54 mg Pb in the rosette leaves). These results confirm that the highest amount of lead accumulated in studied plants stays at the root level as described above but an important translocation of Pb can be observed from the roots to the leaves and even to the siliques.

### Identification of Genes Involved in Lead Tolerance in *H. incana*

In order to characterize the molecular mechanisms involved in Pb tolerance and accumulation in *H. incana*, we identified *H. incana* homologues of several genes previously described in the literature as involved in heavy metal tolerance but not necessarily to Pb, such as *ATM3* (ATP-binding cassette transporter of mitochondrial protein), *CNGC1* (cyclic nucleotide-gated channel), *GS2* (glutathione synthetase), *HMA4* (heavy metal ATPase), *MRP3* (multidrug resistance-associated protein), *MT2a* (metallothionein) and *PCS1* (phytochelatin synthase). A tubulin (*TUB*) gene was identified and used as a reference gene for analysis of gene expression. Partial cDNA sequences were obtained by RT-PCR on leaves or roots total RNA from *H. incana* using primer sequences chosen in the literature or in consensus regions of the corresponding proteins from *A. thaliana* (Table S2). Identification of *H. incana* cDNA sequences was based on the analysis of deduced amino acid sequences, using the BLASTX program. This analysis revealed that all isolated cDNA sequences from *H.incana*, which contained one or more motifs characterizing the corresponding proteins, were highly similar to equivalent sequences from *A. thaliana* (Table 2). On the basis of these 8 genes, the percentage of sequence identities found between *H. incana* and *A. thaliana* was 89% in average with a maximum of 93% for the *PCS1* gene and a minimum of 84% for the *CNCG1* gene in the coding regions. The intergenic transcribed spacer (ITS) region from *H. incana* was cloned and sequenced. The percentage of identity in the ITS region between *H. incana* and *A. thaliana* was 85%. These results verify a close genetic proximity between *H. incana* and *A. thaliana.* This does not mean that the nearest species of *H. incana* was *A. thaliana*. For each gene studied, comparison with available sequences shows that H. incana is also close to *Brassica rapa*, *Noccaea caerulescens*, *Brassica juncea* and *Arabidopsis lyrata* (Figure S2).

**Table 2:** Description of the cDNA sequences isolated from *H. incana*.

Gene name	Length (bp)	Accession^a^	Similarity to *A. thaliana* b
*HiATM3*	407	HQ398196.1	91% to At5g58270
*HiCNGC1*	866	HQ398199.1	84% to At5g53130
*HiGS2*	531	HQ398198.1	88% to At5g27380
*HiHMA4*	863	HQ398195.1	86% to At2g19110
*HiMRP3*	633	HQ398194.1	89% to At3g13080
*HiMT2a*	184	HQ398197.1	88% to At3g09390
*HiPCS1*	118	JF288760.1	93% to At5g44070
*HiTUB*	1066	HQ398200.1	92% to At1g50010

a GeneBank accession number (http://www.ncbi.nlm.nih.gov/genbank/).

b AGI gene code (http://www.arabidopsis.org/).

**Figure 5:**
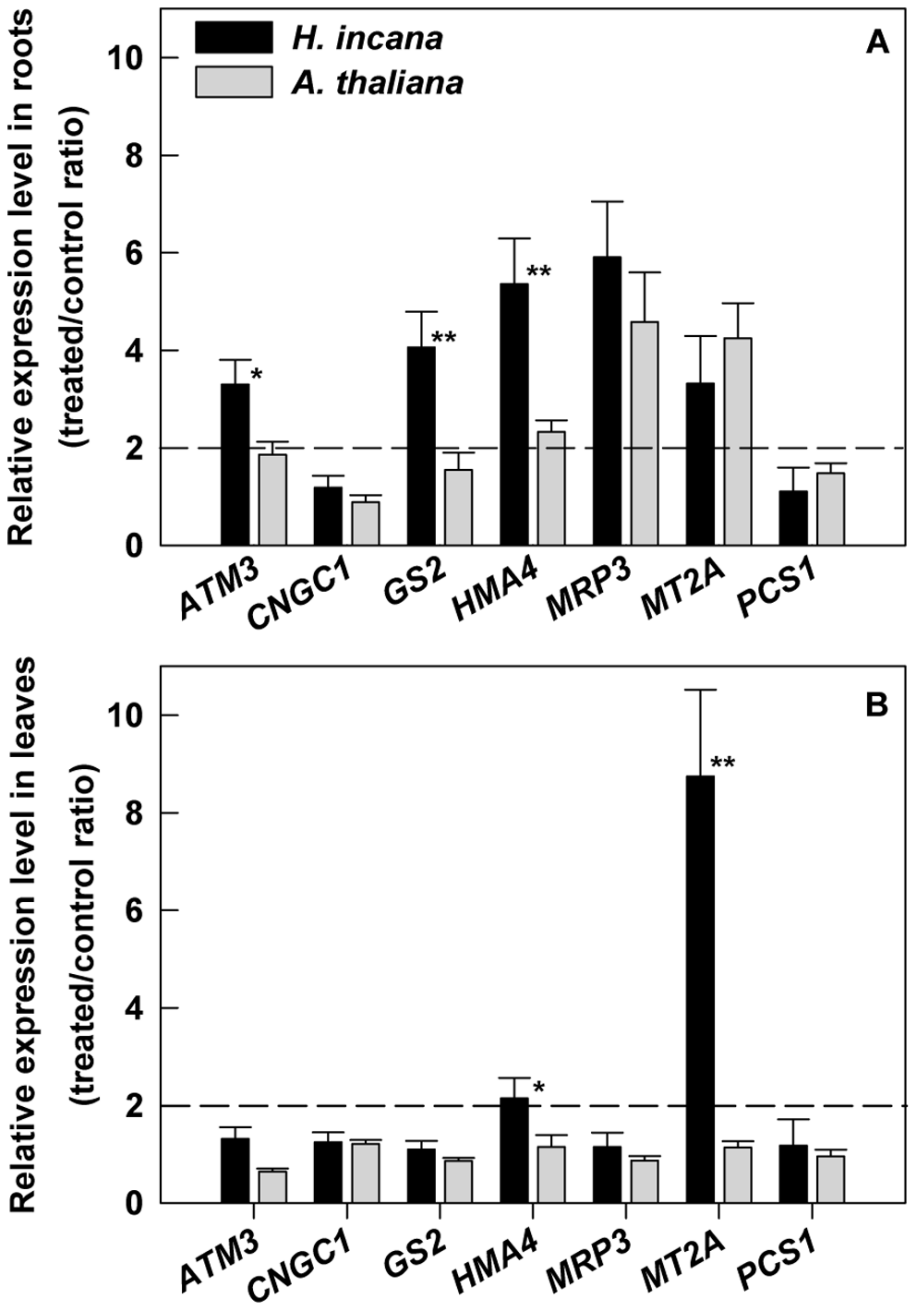
Quantitative analysis of the expression levels of *ATM3*, *CNGC1*, *GS2*, *HMA4*, *MRP3*, *MT2a and PCS1* genes. Expression profiles were obtained from (A) roots and (B) leaves of *H. incana* and *A. thaliana*, Pb-treated or not for 3 days. *ATM3*: ATP-binding cassette transporter of mitochondrial protein, *CNGC1*: cyclic nucleotide-gated channel, *GS2*: glutathione synthetase, *HMA4*: heavy metal ATPase, *MRP3*: multidrug resistance-associated protein, *MT2a*: metallothionein and *PCS1*: phytochelatin synthase. The tubulin gene was used as reference gene and the calibration was done against the roots or the leaves of plants cultivated without Pb. Data are expressed as relative expression level (treated/control ratio) and are the average (± SE) of three independent replicates composed by nine seedlings. * and **Significant difference of values at *p*<0.05 and *p*<0.01, respectively, by Student’s t-test in comparison between *H. incana* and *A. thaliana*. Dashed line indicates a fold change of 2.

The expression profile of the selected genes in response to Pb treatment was determined in *H. incana* and in the non-tolerant *A. thaliana* (Figure 5). Seedlings previously grown on the standard medium were transferred to Pb medium (100 and 40 µM Pb(NO_3_)_2_ respectively for *H. incana* and *A. thaliana*) for a further 3 days. For the selected genes, basic gene expression levels were low in both plants and organs (data not shown). In roots of *H. incana*, an important increase of transcript levels (fold change >2) associated to Pb-treatment was observed for five out of the seven target genes studied: *HiATM3*, *HiGS2*, *HiHMA4*, *HiMRP3* and *HiMT2a* but, among these, only *ATM3*, *GS2* and *HMA4* have a significant expression greater than that obtained in *A. thaliana* (Figure 5A). In leaves of *H. incana*, Pb treatment enhanced approximately by two-fold the expression of *HiHMA4* and more than eight-fold the expression of *HiMT2a* (Figure 5B). The expression of these genes was not significantly increased in Pb-treated plants of *A. thaliana.* These two genes, *HiHMA4* and *HiMT2a*, were particularly affected in both roots and leaves suggesting putative roles in lead tolerance and accumulation in *H. incana*.

**Figure 6:**
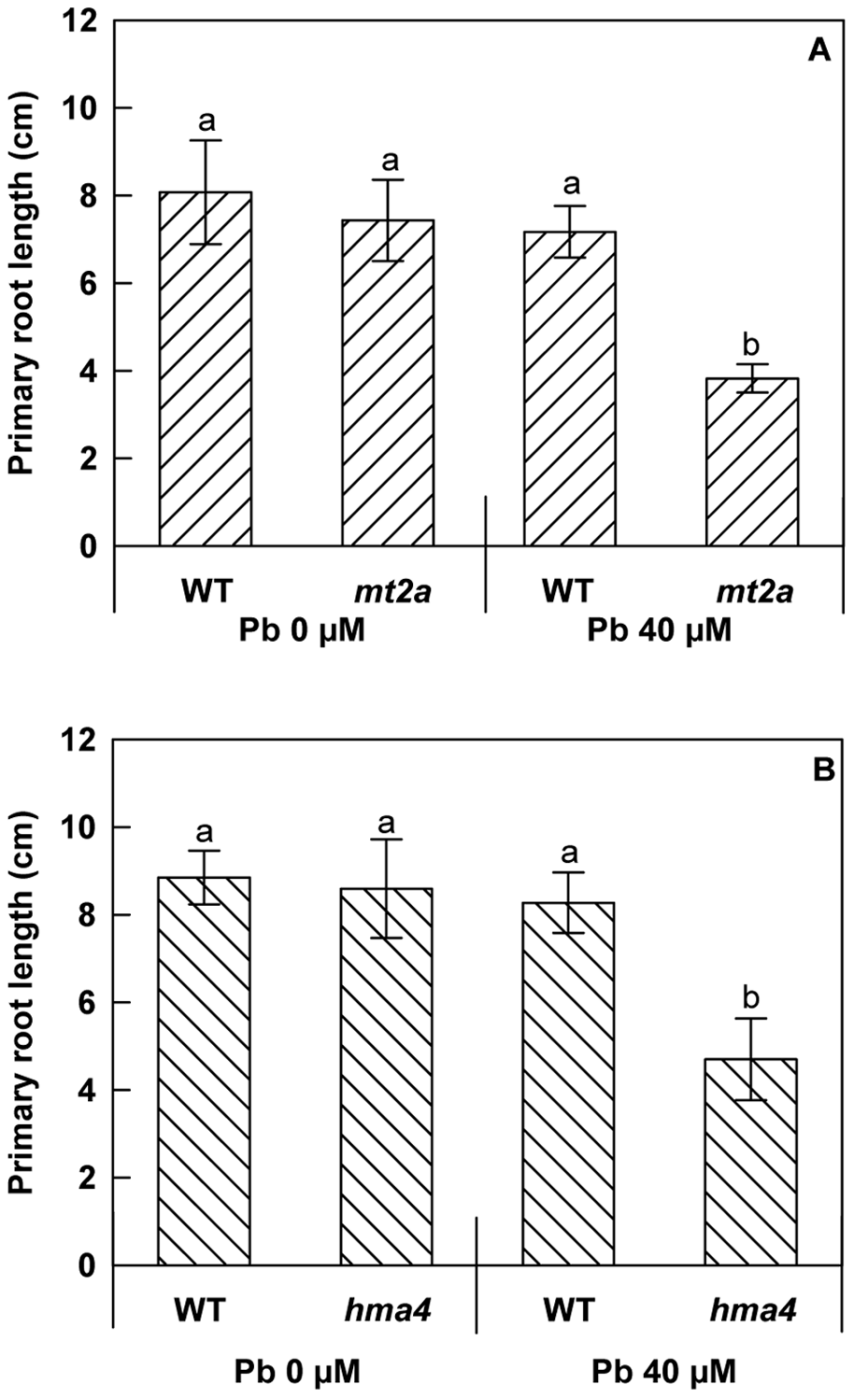
Effects of lead treatment on primary root length in wild-type and knockout mutant seedlings of *A. thaliana*. Seedlings were grown for 13 days on vertically orientated agar plates with or without 40 µM of Pb(NO_3_)_2_. (A) *Atmt2a* T-DNA mutant analysis. (B) *Athma4* T-DNA mutant analysis. All results are the average value (± SE) of 16 seedlings. The letters represent statistically homogenous subgroups using LSD post hoc test at a α = 0.01 significance level.

**Figure 7:**
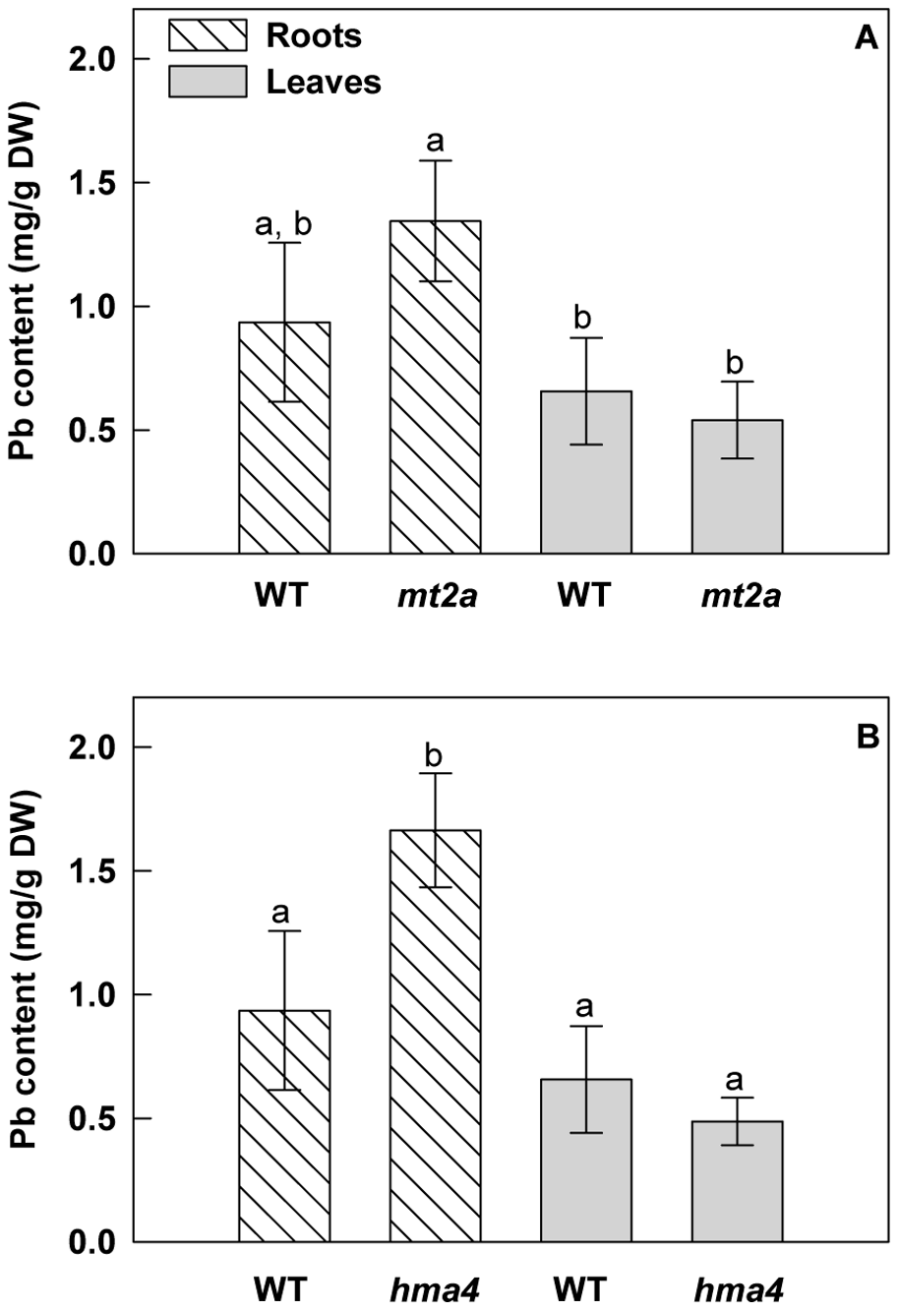
Lead accumulation in roots and shoots of wild-type and knockout mutant seedlings of *A. thaliana*. Seedlings were collected after 13 days of culture on agar plates with or without 40 µM of Pb(NO_3_)_2_. (A) *Atmt2a* T-DNA mutant analysis. (B) *Athma4* T-DNA mutant analysis. All results are the average value (± SE) of three independent replicates. For the roots and leaves separately, the letters represent statistically homogenous subgroups using LSD post hoc test at a α = 0.01 significance level.

If *HiHMA4* and *HiMT2a* are important for lead tolerance, as suggested by their overexpression in the presence of Pb, we expect the corresponding Arabidopsis T-DNA mutants to be more sensitive to heavy metals than wild type plants. To test the physiological function of HMA4 and MT2a in *A. thaliana*, two homozygote lines that contain T-DNA insertion in the *HMA4* gene (Salk_093482, Figure S3) and in the *MT2a* gene (Salk_059712, Figure S4) respectively were isolated from the T-DNA insertion collection generated at the Salk Institute (http://signal.salk.edu). To evaluate the consequences of *HMA4 and MT2a* gene disruption on root growth responses to Pb treatment, we examined the primary root length of mutant plants grown under Pb-treatment and control conditions and found them to be distinguishable from the wild type plants. Wild-type, *hma4* and *mt2a* seeds were germinated directly on standard medium or on medium with 40 µM Pb(NO_3_)_2_. The lead concentration used in this experiment corresponds to the threshold of tolerance that Arabidopsis can withstand in our experimental conditions (data not shown). After 13 days of culture, Pb-treated wild-type plants did not seem to be affected by the Pb treatment compared to the control plants. Similarly when grown on standard medium, both *hma4* and *mt2a* mutants and wild-type plants showed a similar root phenotype. Pb treatment highly significantly reduced primary root length in both *hma4* (45%) and in *mt2a* (48%) mutants (Figure 6). This reduction in primary root growth reflects an increased sensitivity to Pb in the Arabidopsis T-DNA insertion mutants and it suggests that the *HMA4* and *MT2a* genes contribute to lead tolerance. We measured the Pb contents in roots and shoots of the 2 week-old wild-type and *hma4-* and *mt2a-*mutants (Figure 7). Roots of *hma4* plants contained 1.8-fold more Pb than those of the wild-type plants (*p*<0.01). Although the Pb content of *mt2a-*plants seems slightly higher than that of wild-type plants in Pb(NO_3_)_2_-containing medium, the difference was not statistically significant. No effect was observed in Pb content in the shoots. The Pb accumulation observed in roots of *hma4* plants is compatible with an interruption of the translocation resulting from *HMA4* gene disruption.

## Discussion

Unlike Zn and Cu which are essential micronutrients, Pb is non-essential and is detrimental to plant development. Its concentration through the food chain adversely affects biological functions of all living organisms [27]. The identification of genes associated with Pb tolerance and accumulation in plants is the first step towards their application in phytoremediation. Genes conferring Pb tolerance are rare [28] and rarer still are those that confer both Pb tolerance and Pb accumulation.

### *H. incana* is a Lead Accumulator Plant

High tolerance to heavy metals has evolved in a number of plant species leading to a class of rare plants named hyperaccumulators. In the case of Pb the number of plant species able to accumulate this heavy metal in any aboveground tissue in their natural habitat is surprisingly low with a poor degree of diversity [5], [9]. This fact may become an obstacle for the development of phytoremediation projects. The first implementation of phytoremediation strategies is the identification of plant species adapted to environmental conditions and able to accumulate large amount of at least one trace element. For Pb, the most common definition of a hyperaccumulator plant meets the following requirement: the concentration of metal in the shoot must be higher than 1 mg.g^−1^ DW of Pb [4], [8]. Considering this definition, our data obtained from plants collected in tailing mine areas have shown that *H. incana* is a Pb hyperaccumulator (Figure 1). *H. incana* was previously described to accumulate Cu [29], Tl [30] and Zn [31]. Nevertheless, on the polluted site we studied, *H. incana* seems to accumulate preferentially Pb even if Cd, Cu and Zn are present in large amounts in the soil (Figure 1; [24]). The absence of pronounced accumulation of other heavy metals by *H. incana* may be due to competitive interactions between metal ions in these multi-contaminated soils, inhibiting uptake of heavy metals, relative to medium having elevated levels of single metals [31], [32]. In controlled growth conditions on different Pb contaminated soils with no possibility of air-borne contaminations, *H. incana* could accumulate concentration until 3.58 mg.g^−1^ DW of Pb in the aboveground part (Figure 2), confirming the hyperaccumulator trait of *H. incana*. Hydroponic cultures using the bioavailable Pb(NO_3_)_2_ in phosphate free medium is an interesting way to estimate Pb accumulation in both roots and leaves in order to confirm Pb translocation from root to leaves (Figure 3). The amount of lead in leaves after 4 days in hydroponic culture with medium containing 300 µM of Pb(NO_3_)_2_ reaches 0.4 mg.g^−1^ DW. With a longer Pb-exposition, we find lead not only in the stems, the rosette and the stem leaves but also in the siliques (Figure 4). Large differences of lead concentration between root and leaf may indicate an important restriction of the internal transport of this metal from the roots towards the stem, leaves and siliques. However our results rather showed that, although *H. incana* accumulated high concentrations of Pb in the shoots (>1mg.g^−1^ DW of Pb), Pb was mainly stored but not completely immobilized in its roots. Highest concentrations of Pb in roots than in shoots seem to be a general pattern of lead distribution in plants [27], [33], [34]. Taken together, these different experiments demonstrate that *H. incana* could be classified as a Pb accumulator species because it tolerates by far more than 1 mg.g^−1^ DW of Pb in the shoots. However the second criteria defining a hyperaccumulating species, which consists in a higher concentration of the considered heavy metal in the shoots than in the roots, was not fulfilled in *H. incana*. Nevertheless this species implements all the mechanisms that are necessary to translocate Pb from the roots to the aerial parts of the plant and accumulate Pb in different organs.

### *H. incana* is a Powerful Tool to Identify Genes Involved in Pb Tolerance and Accumulation

Several genes previously described in the literature as being involved in heavy metal tolerance and accumulation were selected in order to measure their expression pattern in Pb-treated plants compared to control plants from *H. incana* and *A. thaliana* (Figure 5). Thus among these genes, ATM3 is a mitochondrial transporter that is essential for Fe homeostasis in *A. thaliana* [35] but Kim *et al*. [14] provided several lines of evidence to implicate ATM3 in heavy metal resistance. They showed that the expression of *ATM3* gene is induced either by Cd or Pb and demonstrated that *ATM3*-overexpressing plants grow better than wild-type plants on either Cd- or Pb-containing medium. The activity of *MRP3* promoter from *A. thaliana*, a gene encoding an ABC-transporter, is induced by As, Cd, Cu, Ni, and Pb, but not by Zn and Fe [17]. Disruption by T-DNA insertion mutagenesis of the Arabidopsis gene *CNGC1* conferred plant tolerance to Pb [11]. HMA4 is a Zn/Cd transporter that presented plant tolerance to Cd by loading it into the xylem, thus increasing translocation to the shoot where it might have less damaging effects [13], [36], [37]. Enhanced expression of *HMA4* gene has been shown to be essential for Cd tolerance in Cd-accumulator *A. halleri* [38]. Yeast functional complementation assays revealed that the expression of *MT1a*, *MT2a* and *MT3a* genes in Arabidopsis could increase the tolerance of yeast mutants to Cu and Cd [39], [40], [41]. However, with regard to Pb, little is known about the relationship between the induction of plant MT genes and lead tolerance [42], [43], [44]. It is also the same for phytochelatin that were identified as heavy metal-binding peptides involved in the accumulation, detoxification and metabolism of metal ions (for review [18]). But an alternative to phytochelatins could be glutathione that can also bind lead [19].

In our experiments, gene expression profiles in *H. incana* showed a significant up-regulation (FC>2) by Pb-treatment for some genes such as *HiATM3*, *HiGS2*, *HiHMA4*, *HiMRP3* and *HiMT2a* in roots (Figure 5A) and *HiHMA4* and *HiMT2a* in aerial parts (Figure 5B). The analysis of the expression of these genes in *A. thaliana*, a non Pb tolerant species, showed that only *ATM3, GS2* and *HMA4* were specifically over-expressed in roots and only *HMA4* and *MT2a* were specifically over-expressed in shoots by Pb-treatment in *H incana* (Figure 5). In agreement with previous report, an overexpression of *ATM3* was observed in *H. incana* roots by Pb treatment [14].

From this expression profiling experiment (Fig. 5), two genes, *HMA4* and *MT2a,* retained our attention because they were both specifically over-expressed in roots and/or shoots of *H. incana* compared to *A. thaliana*. The expression of *HiHMA4* and *HiMT2a* was induced two-fold and eight-fold respectively by Pb-exposure in the leaves as well as five-fold and three-fold respectively in the roots. In previous reports *HMA4* gene expression was shown to be modulated by Cd, Mn and Zn treatments [45], [46], and, in the present work, by Pb treatment. The involvement of *HiHMA4* and *HiMT2a* genes in the tolerance process was indirectly studied using T-DNA insertional mutants from Arabidopsis. Two *A. thaliana* mutant lines for respectively *HMA4* and *MT2a* genes were isolated and compared with Col0 wild-type for root growth and Pb content. A significant primary root length decrease was observed under Pb-treatment for both mutant lines, which comforted the implication of these two genes in lead tolerance mechanisms in *A. thaliana* (Figure 6 *)*. Similar approaches were used for the *Athma4* mutant in presence of Zn, Cd and Co but no significant phenotype was found [36], [47] whilst in our case AtHMA4 confers resistance to Pb. In parallel a significant increase of Pb content was observed in the roots and a slight but not significant decrease in shoots of *Athma4* mutants under Pb-treatment (Figure 7). This result can be interpreted as the blocking of translocation leading to an accumulation of Pb in roots and a reduction in shoots. This hypothesis is in concordance with the role of HMA4 in cytosolic metal efflux [6], [13], [48]. In the Cd/Zn hyperaccumulators, *A. halleri* and *N. caerulescens*, HMA4 would be involved in the detoxification of roots by translocated Cd or Zn to the shoots [13], [6]. HMA4 seems to be implicated in lead accumulation in *H. incana* by functioning as metal xylem loading.

For the *MT2a* gene both the up-regulation of the expression by lead exposure in *H. incana* and the phenotype of *A. thaliana mt2a* mutant under Pb-treatment confirmed, for the first time, the implication of a metallothionein in lead tolerance. Several lines of evidence suggest that, as in mammals, plant metallothioneins are involved in the response to oxidative stress and metal toxicity as well as in the regulation of metal homeostasis [49], [50], [51]. In *Festuca rubra*, functional complementation studies using yeast mutant confirmed the functional implication of *MT1* gene in sequestering both essential (Cu, Zn) and non-essential metals (Cd, Pb, Cr) [52]. Recently it was demonstrated that the expression of *AtMT2a* was induced under H_2_O_2_ and low temperature stresses, and AtMT2a functions as an ROS scavenger in the cytosol under abiotic stress conditions [53]. A similar role of MT2a in response to lead exposure can also be suggested.

Taken together, our results demonstrated that *H. incana* is an interesting experimental model to identify new genes involved in the molecular mechanisms of Pb accumulation and tolerance in plants. Indeed its close genetic proximity to *A. thaliana* allows the use of its powerful genetic resources: complete sequencing and annotated genome to identify target genes, T-DNA insertion mutants for functional analysis of these genes.

## Supporting Information

Figure S1 **Phylogenic tree, based on the unweighted pair group method with arithmetic mean, showing the relationships (A) between HiHMA1, AtHMA1 (AT4G37270), AtHAM2 (AT4G30110), AtHMA3 (AT4G30120) and AtHMA4 (AT2G19110), (B) between HiMT2a, AtMT2a (AT3G09390), AtMT2b (AT5G02380), AtMT3 (AT3G15353).** Bootstrap values are indicated in percentage (100 replicates). Multiple sequence alignments were made from the coding DNA sequence using Clustal software.
(PDF)

Figure S2 **Phylogenetic trees based on the Neighbor-joining method showing the relationships between** ***Hirschfeldia incana*** **and the others** ***brassicaceae*** **.** (A) ATM3, *H. incana*: HQ398196, *Arabidopsis thaliana*: NM_125212, *Thellungiella halophila*: AK353402, *Arabidopsis lyrata*: XM_002864513, *Brassica rapa*: AC189355, *Noccaea caerulescens*: AJ746246 and *Populus trichocarpa*: XM_002325589. (B) CNGC1, *H. incana*: HQ398199, *A. thaliana*: NM_124692, *A. lyrata*: XM_002865908 and *P. trichocarpa*: XM_002317724. (C) GS2, *H. incana*: HQ398198, *A. thaliana*: NM_122620, *A. lyrata*: XM_002872228, *B. rapa*: GQ996584, *Oryza sativa*: EU267952. (D) HMA4, *H. incana*: HQ398195, *A. thaliana*: AF412407, *A. halleri*: AY960757, *A. lyrata*: XM_002886195, *N. caerulescens*: JQ904707, *Brassica juncea*: EU418580, *Ricinus communis*: XM_002532190. (E) MRP3, *H.incana*: HQ398194, *A. thaliana*: NM_202570, *A. lyrata*: XM_002884895, *N. caerulescens*: AB162907, *P. trichocarpa*: XM_002300326. (F) MT2A, *H. incana*: HQ398197, *A. thaliana*: NM_111773, *A. lyrata*: XM_002884686, *B. juncea*: Y10850, *B. rapa*: GQ996588, *Brassica oleracea*: AF200712, *N. caerulescens*: FJ439656, *Nicotiana tabacum*: DQ132853. (G) PCS1, *H. incana*: JF288760, *A. thaliana*: NM_123774, *A. lyrata*: XM_002865338, *A. halleri*: AY463694, *B. juncea*: AJ278627, *N. caerulescens:* AY540104, *Triticum aestivum*: AF093752. (H) TUB, *H. incana*: HQ398200, *A. thaliana*: AY149922, *A. lyrata*: XM_002863549, *B. rapa*: DQ414683, *O. sativa*: DQ683569. Multiple sequence alignments were made from the coding DNA sequence using Clustal software.
(PDF)

Figure S3 **Isolation of the** ***mha4*** **T-DNA insertional mutant.** (A) Intron-exon organization of the *Arabidopsis HMA4* gene (At2g19110) and T-DNA location. Solid black boxes and the solid line indicate coding regions and introns, respectively. The position of the T-DNA insertion in the *hma4* allele is indicated by triangle (not to scale). (B) PCR analysis of *HMA4* transcript in wild-type (Col-0) and mutant allele. Expression of *tubulin* (At1g50010) was analyzed as a control. (C) Phenotype of *hma4* mutant seedlings with or without Pb treatment.
(PDF)

Figure S4 **Isolation of the** ***mt2a*** **T-DNA insertional mutant.** (A) Intron-exon organization of the *Arabidopsis mt2a* gene (at3g09390) and T-DNA location. Solid black boxes and the solid line indicate coding regions and introns, respectively. The position of the T-DNA insertion in the *mt2a* allele is indicated by triangle (not to scale). (B) PCR analysis of *MT2A* transcript in wild-type (Col-0) and mutant allele. Expression of *tubulin* (At1g50010) was analyzed as a control. (C) Phenotype of *mt2a* mutant seedlings with or without Pb treatment.
(PDF)

Table S1 **List of the specific primer pairs for quantitative real-time reverse-transcription PCR.** Sequences are listed 5′–3′.
(DOC)

Table S2 **List of the specific primer pairs used for cloning** ***H. incana*** **genes.** Sequences are listed 5′–3′.
(DOC)
