# Supplementary figures and images for: Lead Tolerance and Accumulation in Hirschfeldia incana, a Mediterranean Brassicaceae from Metalliferous Mine Spoils
Source: PLoS One. 2013 May 7;8(5):e61932. doi: 10.1371/journal.pone.0061932 (PMC3646990; doi:10.1371/journal.pone.0061932)

**A**

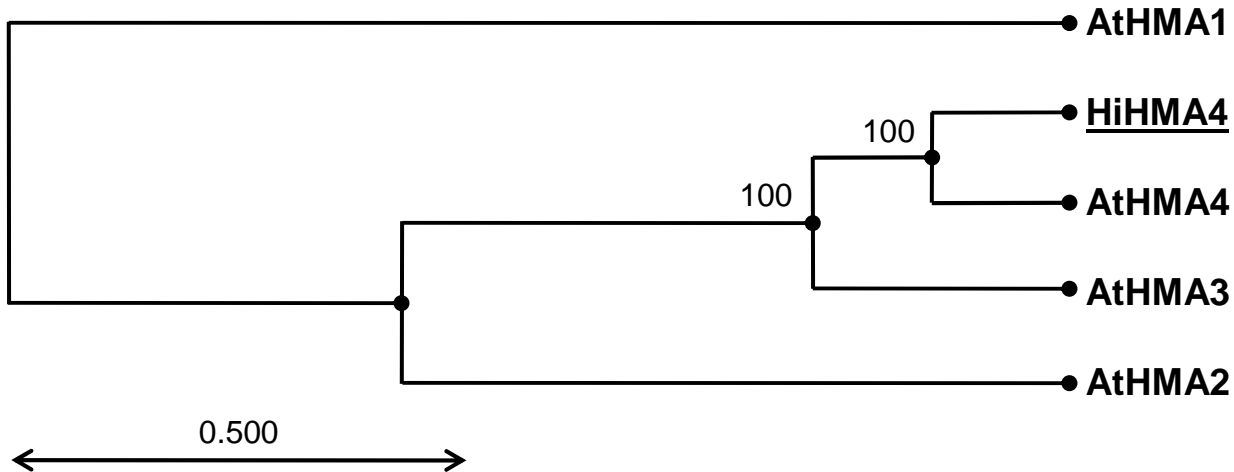

**B**

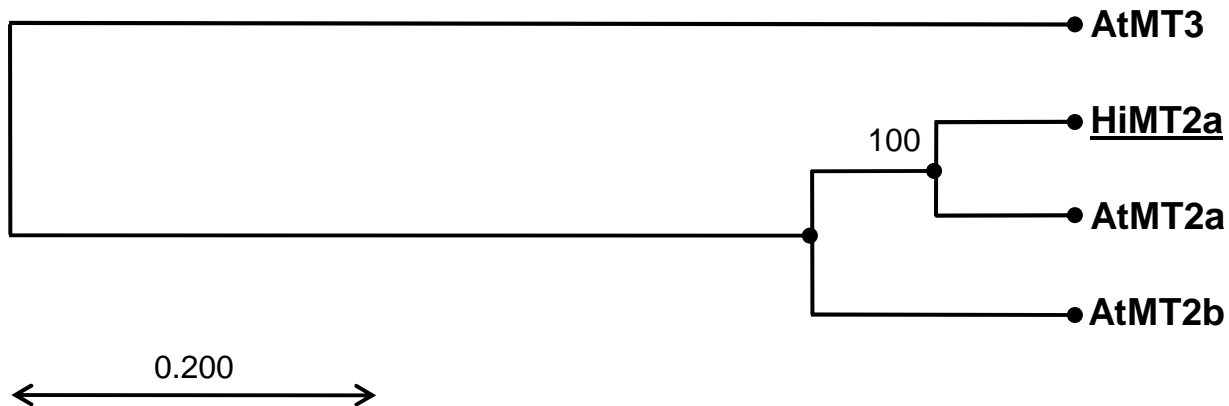

Supplement: Figure S1 — Phylogenic tree, based on the unweighted pair group method with arithmetic mean, showing the relationships (A) between HiHMA1, AtHMA1 (AT4G37270), AtHAM2 (AT4G30110), AtHMA3 (AT4G30120) and AtHMA4 (AT2G19110), (B) between HiMT2a, AtMT2a (AT3G09390), AtMT2b (AT5G02380), AtMT3 (AT3G15353). Bootstrap values are indicated in percentage (100 replicates). Multiple sequence alignments were made from the coding DNA sequence using Clustal software. (PDF) [file pone.0061932.s001.pdf]

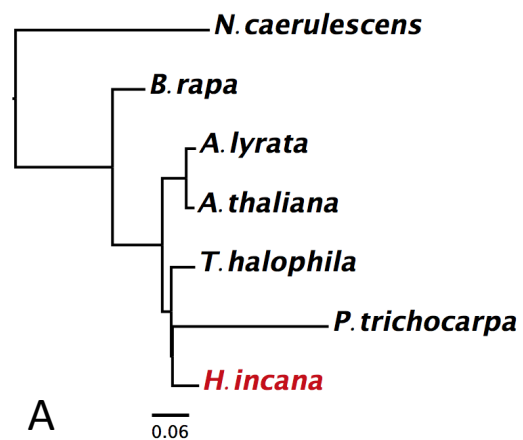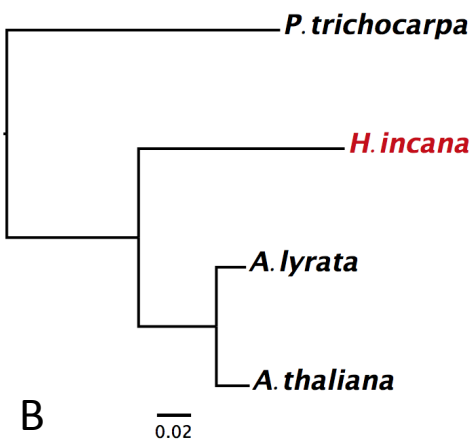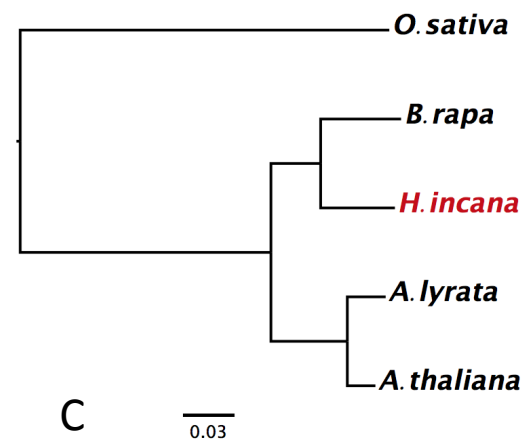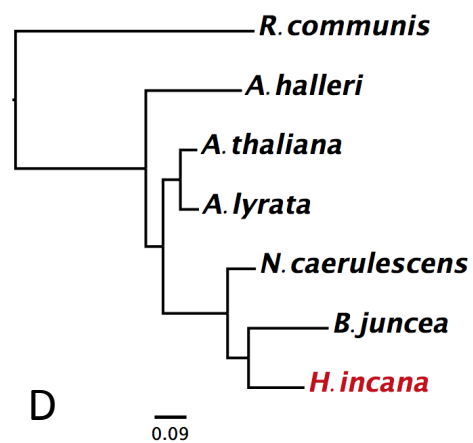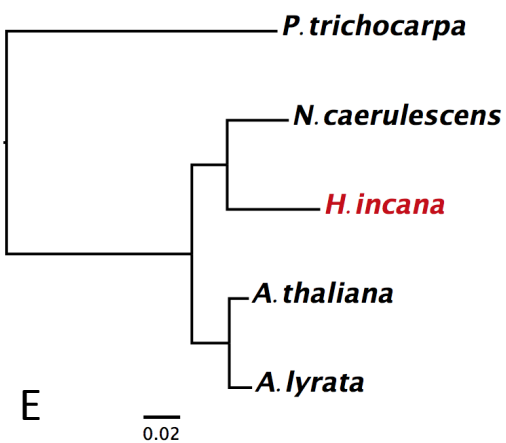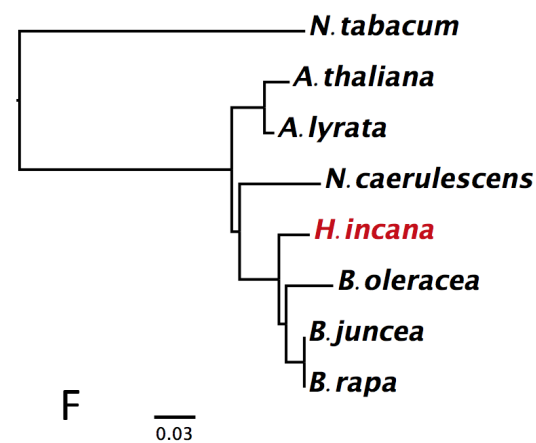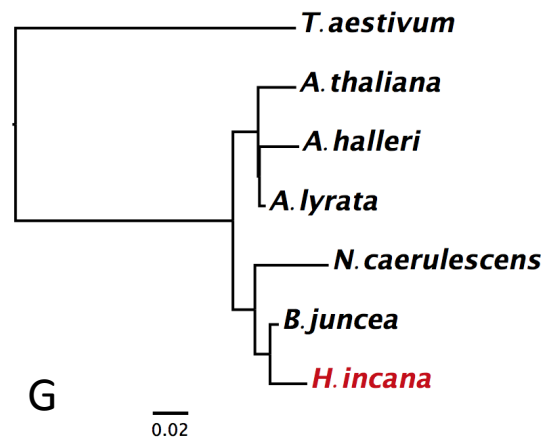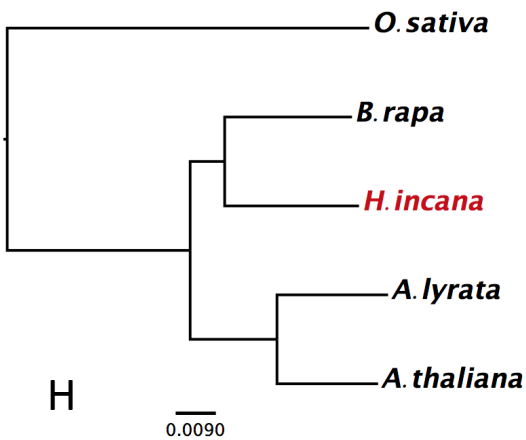

Supplement: Figure S2 — Phylogenetic trees based on the Neighbor-joining method showing the relationships between Hirschfeldia incana and the others brassicaceae . (A) ATM3, H. incana: HQ398196, Arabidopsis thaliana: NM_125212, Thellungiella halophila: AK353402, Arabidopsis lyrata: XM_002864513, Brassica rapa: AC189355, Noccaea caerulescens: AJ746246 and Populus trichocarpa: XM_002325589. (B) CNGC1, H. incana: HQ398199, A. thaliana: NM_124692, A. lyrata: XM_002865908 and P. trichocarpa: XM_002317724. (C) GS2, H. incana: HQ398198, A. thaliana: NM_122620, A. lyrata: XM_002872228, B. rapa: GQ996584, Oryza sativa: EU267952. (D) HMA4, H. incana: HQ398195, A. thaliana: AF412407, A. halleri: AY960757, A. lyrata: XM_002886195, N. caerulescens: JQ904707, Brassica juncea: EU418580, Ricinus communis: XM_002532190. (E) MRP3, H.incana: HQ398194, A. thaliana: NM_202570, A. lyrata: XM_002884895, N. caerulescens: AB162907, P. trichocarpa: XM_002300326. (F) MT2A, H. incana: HQ398197, A. thaliana: NM_111773, A. lyrata: XM_002884686, B. juncea: Y10850, B. rapa: GQ996588, Brassica oleracea: AF200712, N. caerulescens: FJ439656, Nicotiana tabacum: DQ132853. (G) PCS1, H. incana: JF288760, A. thaliana: NM_123774, A. lyrata: XM_002865338, A. halleri: AY463694, B. juncea: AJ278627, N. caerulescens: AY540104, Triticum aestivum: AF093752. (H) TUB, H. incana: HQ398200, A. thaliana: AY149922, A. lyrata: XM_002863549, B. rapa: DQ414683, O. sativa: DQ683569. Multiple sequence alignments were made from the coding DNA sequence using Clustal software. (PDF) [file pone.0061932.s002.pdf]

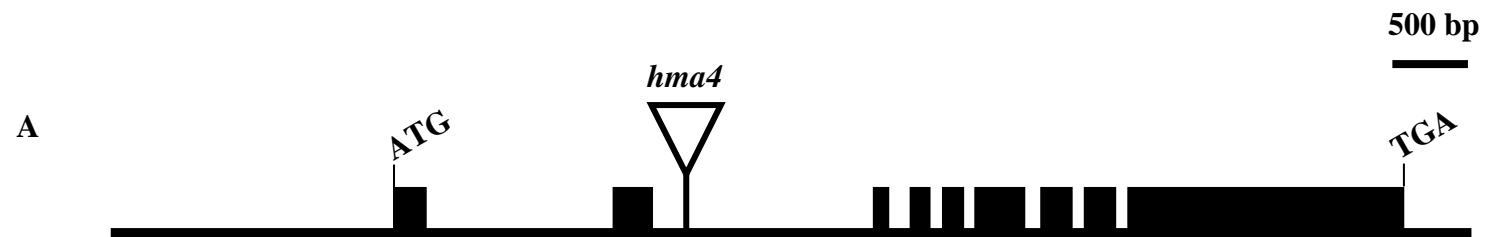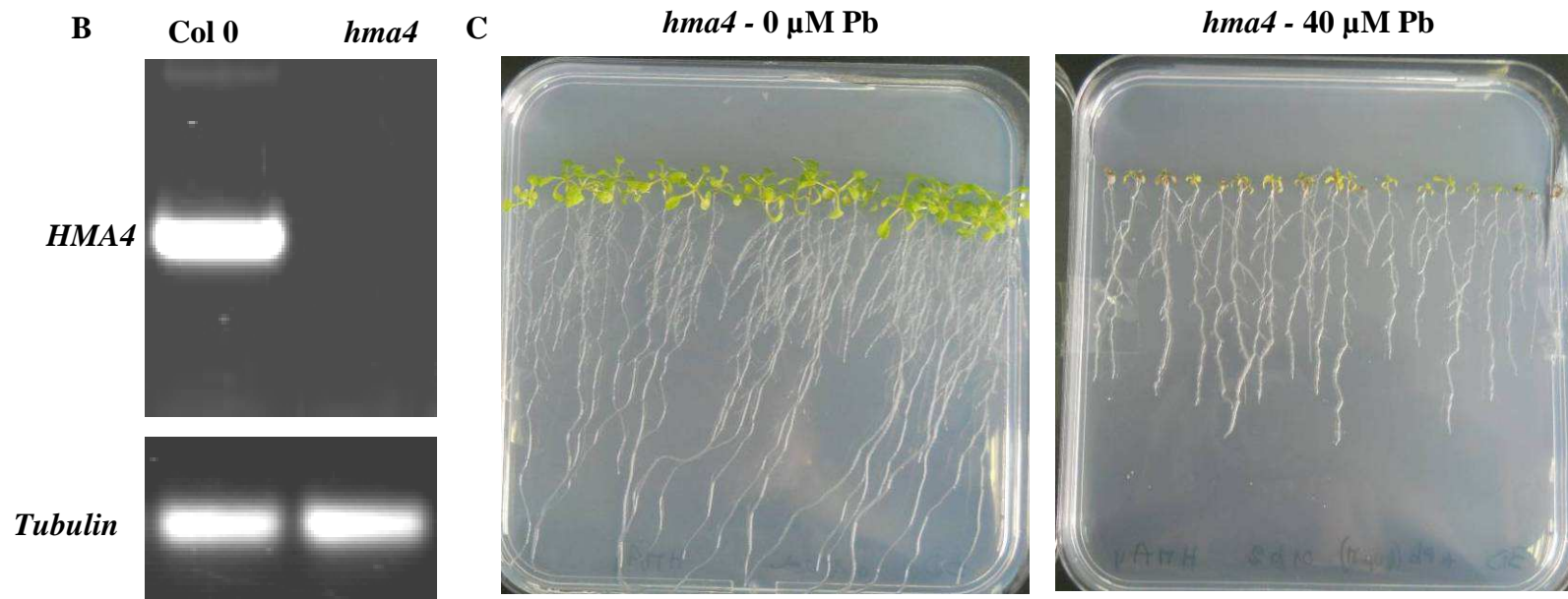

Supplement: Figure S3 — Isolation of the mha4 T-DNA insertional mutant. (A) Intron-exon organization of the Arabidopsis HMA4 gene (At2g19110) and T-DNA location. Solid black boxes and the solid line indicate coding regions and introns, respectively. The position of the T-DNA insertion in the hma4 allele is indicated by triangle (not to scale). (B) PCR analysis of HMA4 transcript in wild-type (Col-0) and mutant allele. Expression of tubulin (At1g50010) was analyzed as a control. (C) Phenotype of hma4 mutant seedlings with or without Pb treatment. (PDF) [file pone.0061932.s003.pdf]

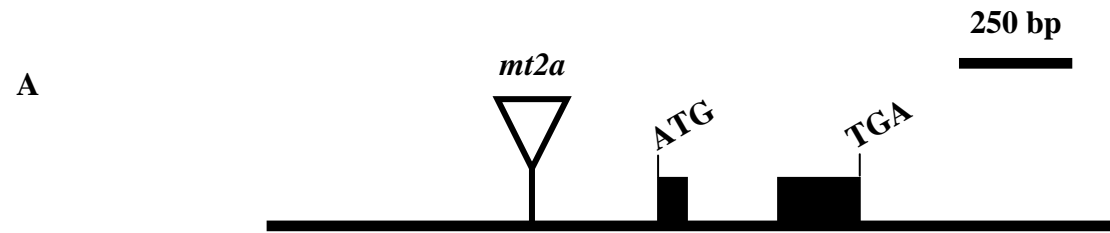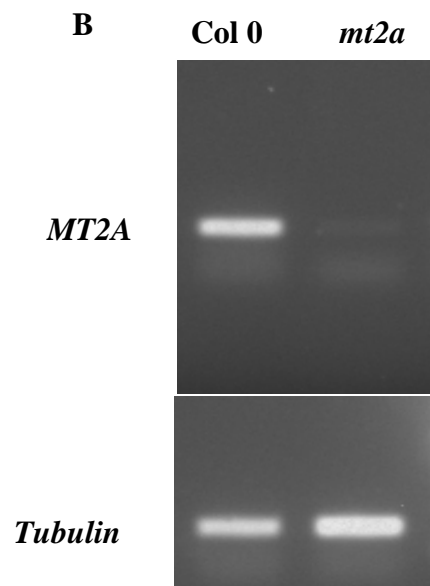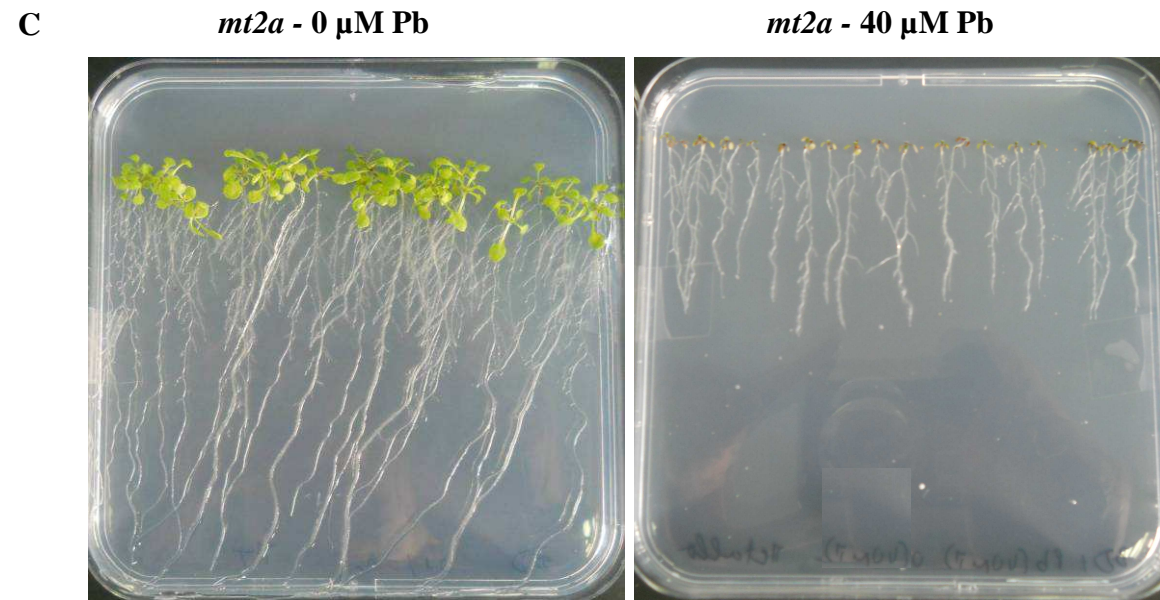

Supplement: Figure S4 — Isolation of the mt2a T-DNA insertional mutant. (A) Intron-exon organization of the Arabidopsis mt2a gene (at3g09390) and T-DNA location. Solid black boxes and the solid line indicate coding regions and introns, respectively. The position of the T-DNA insertion in the mt2a allele is indicated by triangle (not to scale). (B) PCR analysis of MT2A transcript in wild-type (Col-0) and mutant allele. Expression of tubulin (At1g50010) was analyzed as a control. (C) Phenotype of mt2a mutant seedlings with or without Pb treatment. (PDF) [file pone.0061932.s004.pdf]
