# Supplementary material for: Lead Tolerance and Accumulation in Hirschfeldia incana, a Mediterranean Brassicaceae from Metalliferous Mine Spoils
Source: PLoS One. 2013 May 7;8(5):e61932. doi: 10.1371/journal.pone.0061932 (PMC3646990; doi:10.1371/journal.pone.0061932)
Supplement: Table S1 — List of the specific primer pairs for quantitative real-time reverse-transcription PCR. Sequences are listed 5′–3′. (DOC) [file pone.0061932.s005.doc]

**Table S1: list of the specific primer pairs for quantitative real-time reverse-transcription PCR**

Sequences are listed 5’-3’

| **Primer Name** | **Forward primer sequence** | **Reverse primer sequence** |
| --- | --- | --- |
| At_QATM3 | CAGCACAACAGAGGCAGAGA | CACCTTCCCGTTTTCCAGTA |
| At_QCNGC1 | TCACAACAAACGGTGGAAGA | TTGGGAGGTTTGAGGATGAG |
| At_QGS2 | CAAGCAGTCGCAGTGGTTTA | GCCAGTTCTTGCTGGATTTT |
| At_QHMA4 | CACACAGAGCCGTCAAGGTA | TGCATAACTCCTGCAACAGC |
| At_QMRP3 | GTGTTCCCAGTGAACCACCT | CTCGCAACACAAGAGGCATA |
| At_QMT | TCTCCGGCGAGACAACCACAA | TTCTCAGCGTTGTTACTCTCCCCT |
| At_QPCS1 | CGTCAAATGCACGAGTTCTG | GAGGGGGATACTTGAAACG |
| At_QTUB | ATCCACTTCATGCTTTCCTC | GGTAGTTGATTCCACACTTGAA |
| Hi_QATM3 | GTGTGACGAGGCAACAAGTG | CACCTTCCCGTTCTCCAGTA |
| Hi_QCNGC1 | ACAAACGGTGGAAGAACAGG | AACGTATGCCTCAGCTGCTT |
| Hi_QGS2 | CAAGCAGTCGCAGTGGTTTA | GCGAGTTCTTGCTGGATTTT |
| Hi_QHMA4 | CCTCATCTACTTCAACTTCTTCTC | GCAGCAGTCGTGTTCTTATTC |
| Hi_QMRP3 | TTCAAGGGTGGGAGATGAAG | CAAGTGGGATGCCAAGAAGT |
| Hi_QMT | ACTGGTAATGATTCGTTGATG | AGCCTCGTACTGCTGGTTCTTC |
| Hi_QPCS1 | GTATCACAGAGGGGTGTTTA | GAGGGGGATACTTGAAACG |
| Hi_QTUB | ATCCACTTCATGCTTTCCTC | GGTAGTTGATACCGCACTTGAA |
