# Supplementary material for: Lead Tolerance and Accumulation in Hirschfeldia incana, a Mediterranean Brassicaceae from Metalliferous Mine Spoils
Source: PLoS One. 2013 May 7;8(5):e61932. doi: 10.1371/journal.pone.0061932 (PMC3646990; doi:10.1371/journal.pone.0061932)
Supplement: Table S2 — List of the specific primer pairs used for cloning H. incana genes. Sequences are listed 5′–3′. (DOC) [file pone.0061932.s006.doc]

**Table S2: list of the specific primer pairs used for cloning *H. incana* genes**

Sequences are listed 5’-3’

| **Primer name** | **Forward primer sequence** | **Reverse primer sequence** | **Primer origin** |
| --- | --- | --- | --- |
| ATM3 | GCTCGGCGTGCTGCAATTCATG | GCTGCATCAAGCATATCCAC | Modified from Kim *et al*., 2006[14] |
| CNGC1 | GAAGAGACGCAGAACAATGG | CTGGCTCTGCTGGTTTCTGA | Sunkar *et al.*, 2000[10] |
| GS2 | CAAGCAGTCGCAGTGGTTTA | TTCGTCTTTGCTCCTGAGGT | Designed from Arabidopsis |
| HMA4 | AGGTTAGAAGCAAACGTGA | GGTGTAGAGAGGATAAGACC | Designed from sequence alignment |
| MRP3 | caaggacgcacaagtggggag | tgtagaatcctgaaggttgc | Designed from sequence alignment |
| MT | GCATGTGGTTGTGGATCTGG | CACTTGCATTGCATCGTTCTC | Designed from Arabidopsis |
| PCS1 | TGGAGGTGGTTCGATGAATCAATG | GGGATACTTGAAACGAGCAACATC | Dong *et al.*, 2005 [54] |
| TUB | GAGTGCATCTCGATCCACAT | GGTAGTTGATACCGCACTTGAA | Designed from Arabidopsis |
